# Florigen and anti-florigen – a systemic mechanism for coordinating growth and termination in flowering plants

**DOI:** 10.3389/fpls.2014.00465

**Published:** 2014-09-16

**Authors:** Eliezer Lifschitz, Brian G. Ayre, Yuval Eshed

**Affiliations:** ^1^Department of Biology, Technion – Israel Institute of TechnologyHaifa, Israel; ^2^Department of Biological Sciences, University of North Texas, DentonTX, USA; ^3^Department of Plant Sciences, Weizmann Institute of ScienceRehovot, Israel

**Keywords:** flowering time, florigen, mobile growth terminators, SFT/SP regulatory hierarchy, shoot architecture, sympodial tomato

## Abstract

Genetic studies in *Arabidopsis* established *FLOWERING LOCUS T* (*FT*) as a key flower-promoting gene in photoperiodic systems. Grafting experiments established unequivocal one-to-one relations between *SINGLE FLOWER TRUSS* (*SFT*), a tomato homolog of *FT*, and the hypothetical florigen, in all flowering plants. Additional studies of *SFT* and *SELF PRUNING* (*SP*, homolog of *TFL1*), two antagonistic genes regulating the architecture of the sympodial shoot system, have suggested that transition to flowering in the day-neutral and perennial tomato is synonymous with “termination.” Dosage manipulation of its endogenous and mobile, graft-transmissible levels demonstrated that florigen regulates termination and transition to flowering in an *SP*-dependent manner and, by the same token, that high florigen levels induce growth arrest and termination in meristems across the tomato shoot system. It was thus proposed that growth balances, and consequently the patterning of the shoot systems in all plants, are mediated by endogenous, meristem-specific dynamic SFT/SP ratios and that shifts to termination by changing SFT/SP ratios are triggered by the imported florigen, the mobile form of SFT. Florigen is a universal plant growth hormone inherently checked by a complementary antagonistic systemic system. Thus, an examination of the endogenous functions of *FT*-like genes, or of the systemic roles of the mobile florigen in any plant species, that fails to pay careful attention to the balancing antagonistic systems, or to consider its functions in day-neutral or perennial plants, would be incomplete.

## EVOLUTION OF THE FLORIGEN EXPERIMENTAL PLATFORM AND THE SFT/SP REGULATORY PARADIGM IN TOMATO

The florigen hypothesis emerged from elegant grafting experiments in photoperiod-sensitive plants (Chailakhyan, 1936a,b). Extensive experiments in the following 40 years, using a variety of photoperiod-sensitive plants, supported the florigen paradigm and established its core physiological parameters. These were critically evaluated in the superb compendium directed by Zeevaart (1976) and can be summarized as follows: (A) Changing light regimes induces systemic florigenic signals in cotyledons and leaves, which are transported, primarily via the phloem, to the apical meristems, where they induce transition to flowering. (B) While the primary environmental inductive signals may vary, the final stimulus is universal and thus, can be transmitted between species; long-day and short-day plants respond to the same florigenic signal. (C) The florigenic stimulus and the flowering response are quantitative. (D) The florigenic stimulus is balanced by systemic anti-florigenic agents.

In the domains of classic plant physiology, florigen was considered the ultimate and sometimes the sole agent for flowering. The classification of florigen as a systemic stimulant and the prevailing expectation that it is a metabolic product analogous to auxin and other plant hormones, laid the foundation for decades of futile hunts. Florigen took on mythical proportions, and became the “Holy Grail” of plant biology: extensively sought after, but never found (Bernier, 1988; Zeevaart, 2006). With the adoption of genetic and molecular approaches in plant biology, pioneering screens for flowering genes identified five discrete flowering pathways (Koornneef et al., 1991; Simpson and Dean, 2002; Turck et al., 2008). The discovery of seemingly linear, and only partially overlapping genetic pathways, gave rise to post-florigenic interpretations of floral induction, in which florigen had no place. However, further genetic analyses in *Arabidopsis* showed that while several independent flowering inducing pathways exist, the final outputs of all pathways converge on a small number of flowering promoting genes, one notable one being *FLOWERING LOCUS T* (*FT*; Samach et al., 2000; Putterill et al., 2004).

**FIGURE 1:**
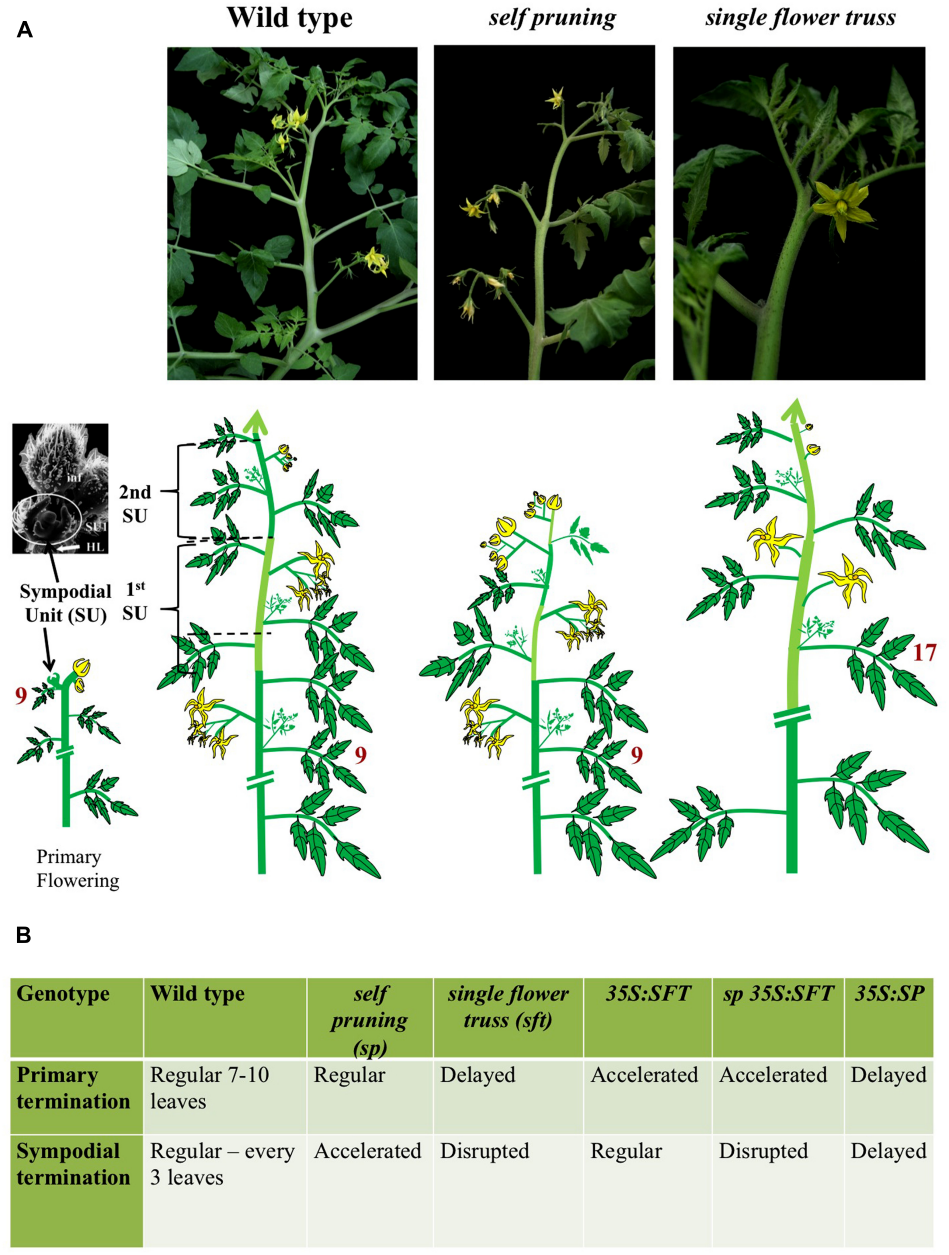
**Genetic regulation of shoot architecture in sympodial tomato. (A)** Live images (top) and the corresponding schematic illustrations (bottom) of the wild type, *self-pruning* and *single flower truss* shoots of tomato. The primary shoot of wild type tomato (left) is terminated by the first inflorescence relatively early, after about 8–12 leaves. The first termination event activates the sympodial cycle (bottom left). In sympodial plants, the apparent main shoot consists of a reiterated array of sympodial units (SU). Each SU arises from the most proximal axillary meristems, and, depending on the prior regulated termination of the preceding unit, consists, in the case of tomato, of three vegetative nodes and a terminal inflorescence. The tomato inflorescence itself is a sympodial structure in which vegetative nodes are suppressed, intercalary leaves removed and each SU is terminated by a single flower. Similarly, the complexity of SUs may vary between systems being reduced to two vegetative nodes in cotton or one in Petunia. The “determinate” shoot of the *self-pruning* mutant plants (middle). A mutant *sp* gene accelerates the termination of SUs but does not change the rules of the sympodial habit as such. The result is a progressive reduction in the number of vegetative nodes between inflorescences in a pattern that depends on light intensity and genetic background. The indeterminate growth in *sft* mutant plants (right). Unlike *SP*, the *SFT* gene targets, among its other pleiotropic functions, the sympodial branching pattern proper. In *sft*, primary termination is delayed and the terminating organ forms one flower and then proceeds as a vegetative shoot with irregular intercalary flowers. And since the formation of a new SU depends on the termination of the former one, the incomplete termination in *sft* results in the delayed formation, or complete suppression of the sympodial buds. Axillary buds release form leaves of the primary shoot or vegetative inflorescence follow the same path to generate the *sft* shoot system. Note that no full-proof loss-of-function allele of *SFT* is available and that the number of leaves formed by the vegetative inflorescence depends on the genetic background, and on the integral light doses. **(B)** Dose-dependent regulation of primary and sympodial termination by the *SFT* and *SP* genes. The SFT/SP paradigm is derived from the contrasting, but at the same time mutually dependent effects, of the loss and gain of functions of the two CETS genes. For examples: *sft* disrupts the sympodial habit but is epistatic to *sp* in the double mutant combination. The effects of *SFT* on the sympodial habit, stem growth, and leaf complexity depend on the dose of *SP* and in general the morphogenetic effects of mutant SP depend on a functional *SFT.*

Our encounter with the florigen odyssey was stirred by the analysis of *SELF-PRUNING* (*SP*) in tomato (Yeager, 1927; MacArthur, 1932; Pnueli et al., 1998), a perennial, day-neutral bush with a stereotypical sympodial growth habit (**Figure 1**). The upright growth of tomato is manifested by an apparent linear shoot consisting of consecutive sympodial units (SU), each forming three leaves before terminating in a compound inflorescence. Mutant *sp* plants form progressively shorter SUs, until the shoots terminate in two successive inflorescences (**Figure 1**). Therefore, in wild type (WT) and in *sp* plants, flowering is synonymous with termination (i.e., termination of the SU), with *SP* functioning as an anti-terminator, maintaining vegetative growth (i.e., production of leaves) in each SU. This basic understanding inspired the appreciation that “termination” is the prime function of florigen. In retrospect, *SP* was the first annotated flowering antagonist gene and the introduction of the recessive *sp* gene into tomato cultivars 70 years ago, facilitated mechanical harvesting, industrial production, and the irreversible flooding of the world with ketchup.

The identification of *CENTRORADIALIS* (*CEN*) of *Antirrhinum majus* and *TFL1* of *Arabidopsis* as homologous genes that maintain the indeterminate habit of the shoot apical meristems (SAMs) of monopodial plants (Bradley et al., 1996, 1997), conceptually and practically facilitated the cloning of *SP* as the third member in the *CETS* gene family (Pnueli et al., 1998). These findings were followed by the cloning of *FT*, another CETS-family member (Kardailsky et al., 1999; Kobayashi et al., 1999). Then, critical evidence was obtained that the proteins produced by *SP* of tomato and *FT* of *Arabidopsis* share binding partners (Pnueli et al., 2001) and that the tomato homolog of FT is the late-flowering *SFT* gene (Lifschitz et al., 2006). In contrast to *SP*, inactivation of *SFT* suppresses termination, consequently promoting the formation of an indeterminate vegetative inflorescence, typically consisting of one or a few flowers intervened by leaves. Furthermore, because the release of new SUs is linked to termination, *sft* concomitantly suppresses the timely formation of SUs (**Figure 1**). By regulating the periodicity of vegetative–reproductive switches, *SP* and *SFT* dictate the overall architecture of the shoot system.

**FIGURE 2:**
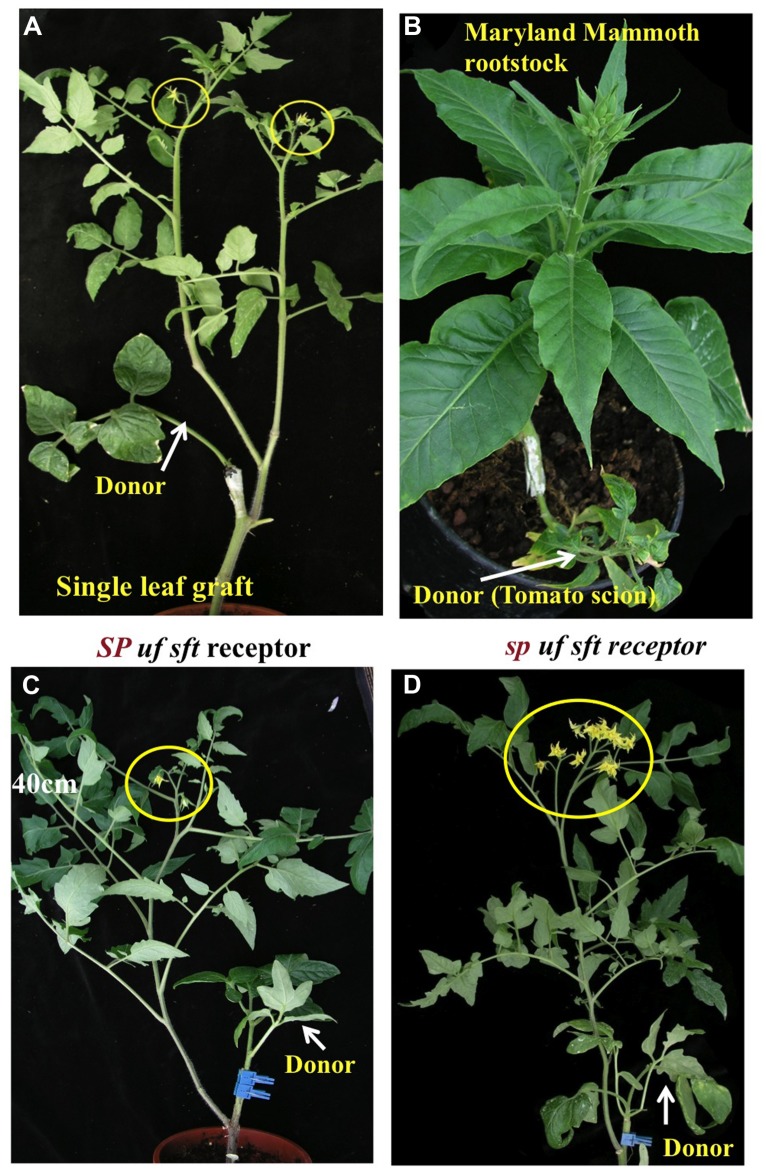
**Systemic delivery of Florigen and its local check by SP. (A)** A single leaf donor can stimulate flowering in an ever-vegetative *uf sft* recipient. **(B)** A Tomato florigen donor induces flowering in a long-day grown MM tobacco plant (Lifschitz et al., 2006). **(C,D)** Quantitative amelioration of Florigen impact – the regular response of ever-vegetative *uf sft* recipient shoot **(C)** is greatly enhanced in the absence of *SP* **(D)**.

Thus, genes promoting termination and flowering in monopodial, indeterminate, and photoperiod-sensitive plants congregate under a single molecular umbrella together with genes regulating the growth/termination cycling in perennial day-neutral plants. The evidence for a common molecular flower-promoting denominator in such diverged systems, and the fact that of all genes assigned to the multiple flowering pathways, only *FT/SFT* were not transcription factors, expedited our attempts to duplicate the grafting experiments that evoked the florigen hypothesis. Instead of exploiting donors induced by photoperiodic signals, we used *SFT*-overexpressing tomato plants, and instead of receptors/testers growing under non-permissive day-length conditions, we used tomato lines defective in flowering genes. These experiments showed that *SFT* generates graft-transmissible signals that substitute for external and internal flowering signals and established, for the first time, an unequivocal one-to-one relation between the elusive florigen and a single Mendelian gene (**Figure 2**; Lifschitz et al., 2006). The requirements for universality were satisfied by the mobile florigen-complementing flowering mutants independent of light regimes and were further substantiated by demonstrating that a tomato donor of *SFT* rescued flowering of the classic Maryland Mammoth tobacco grown under non-permissive conditions (Garner and Allard, 1920; Yang et al., 2007). The genetic evidence for the florigenic signal being a protein, and not RNA, is convincing and amply discussed (Lifschitz et al., 2006; Kobayashi and Weigel, 2007; Lin et al., 2007; Mathieu et al., 2007; Turck et al., 2008), although critical details of the systemic pathway as discussed below, are still lacking or under debate.

Gene dosage analysis and epistasis tests using loss- and gain-of-function *SFT* and *SP* genes, revealed that, in addition to their effects on flowering, the two genes are involved in multi-organ pleiotropic effects, all of which can be traced to shifts in the growth-termination equilibrium. Concomitantly, grafting experiments showed that every developmental change conditioned by endogenous shifts in the SFT/SP ratios can be reproduced by elevated ratios imposed by graft-transmissible florigen (Shalit et al., 2009).

Integration of the results from complementary grafting and genetic experiments provided a new working hypothesis for the florigen world: endogenous SFT/SP ratios regulate local growth-termination equilibria in all meristems of the tomato shoot system. A switch from growth to maturation and termination is triggered by an organ-specific shift from a low to a high SFT/SP ratio. In its refined version, and within given thresholds, the SFT/SP paradigm specifies that the mere shift, rather than the absolute levels of its components, determines the developmental outcome. Florigen originates primarily in mature leaves, which are the best exporters of florigen, but is distributed to all organs including other developing leaves. Thus, by enhancing the maturation of acceptor leaves, florigen generates an autoregulatory systemic information system. Florigen functions as a universal growth hormone (Shalit et al., 2009; Turnbull, 2011), where its role in boosting flowering reflects its fundamental function as a modifier of growth and termination across the shoot systems. In all its roles, the florigen protein emerges as both the architectural regulator and “great communicator” of the shoot systems in flowering plants.

Below, we review the impact of the mobile florigen in the context of the SFT/SP paradigm, on development, and while many pioneering molecular details associated with the endogenous functions of *FT*-like genes were formulated in *Arabidopsis*, we use tomato as the “centre of gravity” in this review.

## FLORIGEN, FLOWERING PATTERNS, AND SHOOT ARCHITECTURE

Shoot and flowering systems in plants differ in their inherent potentials to respond to environmental signals and in their innately regulated growth habits, which together dictate different adjustments of, and by, the florigen system. Comparison of the florigen systems in different species facilitates the classification of its universal versus species-specific functions; however, such a comparison is only useful when the growth habits of the examined plants are well understood. In their response to photoperiods, plants are categorized as long-day, short-day, or day-neutral plants. Species can be classified by their life cycles as annual, biennial, or perennial, each with its own adaptive florigenic system, and also by their growth habits as monopodial or sympodial. In the biennial onion, the response to floral signals depends on exposure of the bulb to a cold winter (Lee et al., 2013), whereas in the carrot, storage is built up during the first year and flowering occurs in the following summer. In short-lived perennials such as *Arabis alpina*, flowering is restricted to particular branches that will flower in the next year, only after exposure to inductive conditions (Wang et al., 2011). Interestingly, a short-lived perennial, or alternatively, an annual habit, is observed in populations of monkey flowers living in near-shore habitats or in more inland populations, respectively (Lowry and Willis, 2010). Polycarpic perennial bushes with multiple flowering cycles in every growing season, such as tomato, maintain a sympodial habit in all their shoots (**Figure 1**), whereas others, like cotton, display a blend of developmentally regulated sympodial and monopodial shoots (Mauney and Ball, 1959; McGarry and Ayre, 2012a,b). Deciduous perennials, such as apple, maintain largely sympodial branching and regulate flowering in lateral branches by a combination of endogenous and external cues (Hallé et al., 1978; Costes and Guèdon, 2012).

How are the different components of the florigen system, which includes biosynthesis, cellular compartmentalization, export, phloem transport, and targeting, adapted to regulate such diverse patterns of vegetative and reproductive cycles?

The annual monopodial and day-length-sensitive plants are best represented by *Arabidopsis* (Meyerowitz, 1989; Koornneef et al., 1991). The apical meristem of *Arabidopsis* is indeterminate throughout its life cycle and all appendages are laterals, with leaves and flowers being the only determinate organs. The annual life cycle of *Arabidopsis* must match the seasons, and requires a timely, and thereby inducible, transition to flowering. This occurs once during the life cycle, affects both apical and axillary apices and is manifested by a clear distinction between vegetative and reproductive phases. Accordingly, in order to serve its role in flowering, florigen may reach the primary apex only once in a lifetime, within a narrow time window. Such an initial day-length signal is mostly sufficient to activate flowering in lateral shoots (Corbesier et al., 1996).

The inducible flowering systems of annual plants, such as *Arabidopsis* and rice, are also regulated, in part, by internal signals, but primarily by signals generated by light quality and periodicity (Putterill et al., 2004). Central to the upstream activating program of *FT*-like genes in photoperiod-sensitive annual plants, is the circadian clock output transmitted by the *GIGANTEA-CONSTANS* (*GI-CO*) pathway (Suarez-Lopez et al., 2001; Mizoguchi et al., 2005). CO lacks a DNA-binding domain and is recruited to CCAAT binding sites of *FT* by NF-YC, a member of the trimeric CBF family (Ben-Naim et al., 2006; Wenkel et al., 2006; Kumimoto et al., 2008). The role of CO in regulating *FT* in day-length plants was adequately demonstrated by its contrasting effects on the *FT* genes of *Arabidopsis* and rice (Putterill et al., 1995; Hayama et al., 2003), but variations on these themes have been reported. For example, expression of tomato *CO* failed to modify flowering time in tomato, *Arabidopsis*, and tobacco (Ben-Naim et al., 2006). In the short-day *Pharbitis nil*, the daily expression profiles of the two flowering-promoting *FT* paralogs are uncoupled to those of *CO*, suggesting that *FT* in this species might be regulated by other transcription factors (Hayama et al., 2007). Therefore, mechanistic claims based solely on expression profiles of the seasonal CO-FT module in other plants, particularly deciduous trees, should be considered with caution because, unlike in *Arabidopsis* and *Pharbitis*, such claims have not been backed by rigorous genetic tests (Ballerini and Kramer, 2011). Direct conditional repressors of *FT* in *Arabidopsis* include the MADS genes *FLC* and *SVP* (Li et al., 2008), *SMZ* (Mathieu et al., 2009), *PIF4* (Kumar et al., 2012), epigenetic transcription regulators (Turck et al., 2007; Adrian et al., 2010), and more. But because florigen activates flowering in response to multiple environmental and endogenous signals, regulatory relations discovered in *Arabidopsis* should only be taken as a lead for system-specific studies.

## THE TOMATO SYMPODIAL SHOOT SYSTEM AND THE SFT/SP RATIO

The sympodial system of tomato, with its polycarpic and polycyclic habits, captures the most typical features of deciduous perennials, such as grape vines and apple trees, or of perennial bushes like roses, cotton, potato, or black nightshade (*Solanum nigrum*; Bell, 1992). The sympodial shoot system of tomato is a conglomerate of three branching habits: (A) Axillary meristems of the primary shoot, which are first arrested by apical cues but are gradually released from dormancy after floral termination of the apical meristems above them (**Figure 1**). (B) Sympodial branching, involving an axillary meristem, hosted by the third leaf of each SU that is not subjected to apical dominance and grows out in response to signals generated by the terminating SAM, with no intervening dormancy to form the next SU (Lifschitz and Eshed, 2006). When the termination of the sympodial apex is delayed or accelerated, the formation of the next SU will be affected in a similar manner. Thus, “termination,” floral transition, phyllotaxis, and branching in each SU must be coordinated in the apical bud, within a distance of 10–50 cells. (C) Regulated branching of the two remaining basal axillary meristems of each SU, from which only the second is regularly activated after being released from apical dominance. Because each SU is a replicate of its predecessor, their formation requires a self-perpetuating mechanism, with regularly cycling flowering and anti-flowering messages. Notably, the inflorescence shoot itself is a sympodium from which nodal leaves are removed and in which each flower represents the terminating organ of the previous SU (Lifschitz and Eshed, 2006; Lippman et al., 2008; Park et al., 2014; Périlleux et al., 2014).

## SFT/SP RATIOS IN SHOOT MERISTEMS

In the tomato apex, while *sp* accelerates termination of SU in a progressive, age-related manner, the primary shoots of *SP* and *sp* isogenic plants terminate after the same number of leaves. Conversely, overexpression of *SP* delays flowering of both primary and sympodial apices (Pnueli et al., 1998). A similar delay in the termination of the primary apices occurs in *sft* plants and results in an indeterminate inflorescence shoot with a mixture of leaves and solitary flowers that substitute for the regular compound inflorescence. Overexpression of *SFT* induces extreme premature termination of the primary shoot, but, in contrast to *sp*, the shoot continues to form regular SUs. When *SFT* is overexpressed in the *sp* background, the sympodial system collapses, as manifested by termination of the primary apex with only 1–3 flowers, arrest of the sympodial meristems and their failure to support the formation of both a compound shoot, and formation of 1–3 leaves on the 3–4 axillary meristems of the primary shoot before terminating with a “blind” apex (Shalit et al., 2009). Termination induced by high SFT concentrations is therefore sensitive to *sp*, particularly in the SUs, which are resistant to high SFT levels under functional *SP*.

Termination and flowering in cultivated tomato are not sensitive to day length but are extremely sensitive to integral light doses (Kinet, 1977). Under low light conditions, primary flowering is delayed: first inflorescences tend to abort at their primordial stage, mature inflorescences turn partial leafy, and flowering within the SU is extended from three to five or six leaves. *sp* plants are less sensitive to low light intensity, while *sft* plants are much more sensitive than their WT siblings. Overexpressors of *SFT*, in WT or *sp* backgrounds, are virtually insensitive to low-light intensities. Likewise, *sp* accelerates sympodial of flowering in every examined recessive late-flowering background. Therefore, both *sp* and high SFT/SP ratios can substitute for light in tomato.

In agreement with their responses to the SFT/SP ratio, the sympodial meristems are also sensitive to intermediate levels of SP and SFT. When *SFT* is expressed in *sp/+* heterozygous plants, regular SUs, with two instead of three leaves, dominate the shoots (Shalit et al., 2009). But the reproductive differentiation of the shoot apex is also sensitive to dose changes in *sft/+* plants. Krieger et al. (2010) reported that tomato plants heterozygous for *SFT* and growing under wide spacing conditions, produced a much higher fruit yield as compared to other heterozygous lines, and attributed this effect to a single gene heterosis. More recently, dedicated measurements and appreciation of the fact that the heterozygous plants were also homozygous for *sp*, led to the conclusion that a dosage response to SFT, i.e., the SFT/SP ratio, tunes shoot architecture in a quantitative manner, and in particular field stands, such tuning may result in higher yields (Jiang et al., 2013).

Primary and sympodial tomato meristems are, as mentioned above, differentially sensitive to similar SFT/SP ratios. In the mature inflorescence shoots of monopodial *Arabidopsis*, buds in the axils of bracts terminate with a flower, with no intervening vegetative phase, while the primary SAM remains indeterminate. The SAM is “protected” from florigen activity by *TFL1*, as shown by *tfl1* primary shoots, which are terminated by a flower while their flowering time is only marginally affected (Shannon and Meeks-Wagner, 1991; Alvarez et al., 1992). However, lateral shoots of *tfl1* plants flower after the formation of 0–2 leaves instead of 2–5 leaves in WT. Therefore, flowering and the role of SFT/SP (FT/TFL1) ratios in both annual *Arabidopsis* and perennial tomato, must be considered in the framework of the two flowering systems, one for the primary shoots and the other for lateral shoots, sympodial in tomato and the regular laterals in *Arabidopsis* (Lifschitz and Eshed, 2006).

Importantly, all meristematic activities in the shoot systems can be terminated via elevated SFT/SP ratios. In particular, arrest of lateral expansion and secondary growth of the stems, most likely by attenuated cambial activity, typical to *SFT* overexpressing plants, is maintained throughout growth even if the sympodial cycle is completely recovered. Under the same SFT level, as noted above, sympodial meristems of the same shoots, maintain a regular, even if accelerated, 3-leaf cycle. The quantitative, meristem-specific impacts of the SFT/SP ratio are further illustrated through the response of the compound leaf to high SFT/SP ratios.

## COMPOUND LEAVES VIEW THE 1:1 RATIOS OF *SFT/SP* AND *sft/sp* AS EQUALLY INFORMATIVE

**FIGURE 3:**
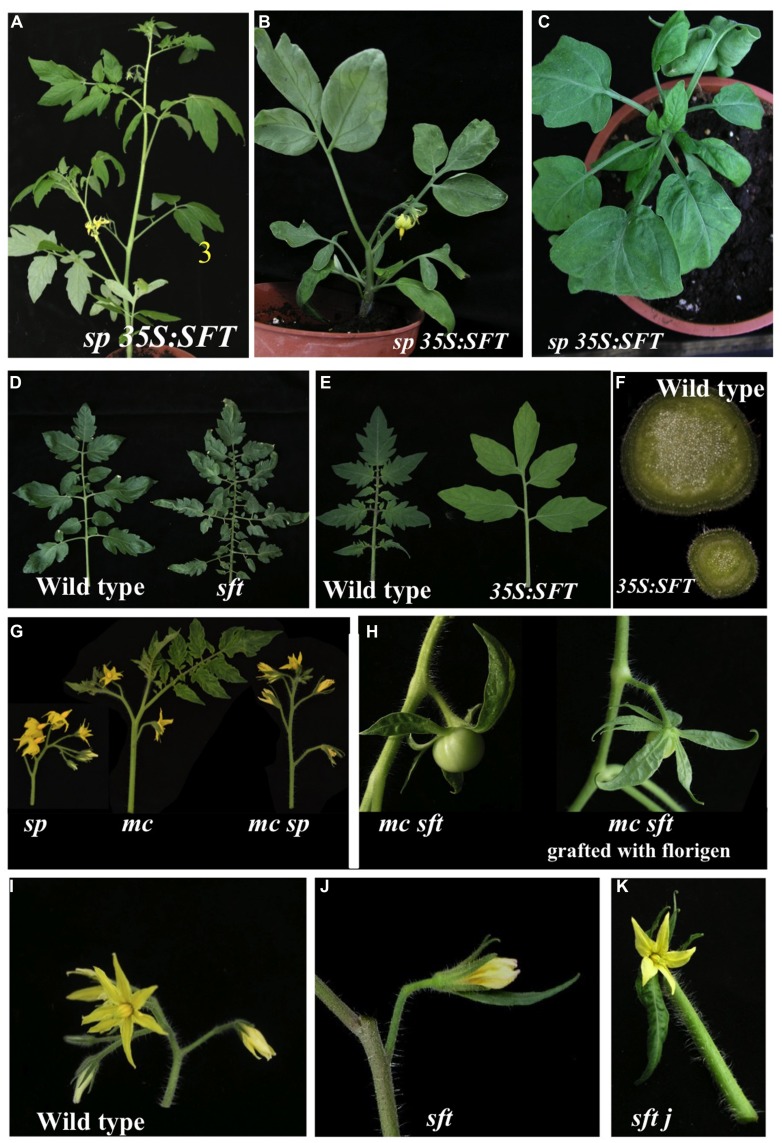
**Diverse developmental systems are regulated by the SFT/SP ratio. (A)** Tomato plants expressing the *35S:SFT* gene flower very early but maintain normal sympodial cycling. **(B,C)** In the absence of *SP* high SFT suppresses axillary meristems **(B)** or even vegetative and inflorescence apical meristems. **(D)** *sft* leaves generate extra leaflets and folioles **(E,F)** *35S:SFT* plants produce simpler leaves with entire margins. **(E)** High SFT induces thinner stems by arresting secondary growth. **(G)** Loss of *MACROCALYX* (*mc*, a putative homolog of *AP1*) results in partially leafy inflorescence that is rescued by further loss of *SP*. In contrast, the incomplete abscission zone of *mc* is completely eliminated in *mc sft* but is fully restored by a mobile florigen **(H)**. **(I–K)** Within the flower, *sft* **(J)** conditions the formation of a larger adaxial sepal which is greatly enhanced in *jointless1* (*j1*) background **(K)**.

A correlation between leaf growth and flowering has been frequently observed in many plants but has generally been attributed to secondary effects of flowering. Our results implied that transition to flowering and reduced leaf-growth represent two facets of the same developmental process. The formation of a compound leaf in tomato requires the activation of lateral leaflet meristems along the primary rachis and of additional rounds of ramification along secondary leaf rachises (Hareven et al., 1996; Efroni et al., 2010). The complexity of the leaf is marginally reduced in *sp* plants, but plants overexpressing *SP*, e.g., bearing a low SFT/SP ratio, show excessive activity of the plate meristem in the lamina (Shalit et al., 2009). *sft* leaves have excess intercalary leaflets (folioles, Molinero-Rosales et al., 2004), while leaves overexpressing *SFT* form smaller blades and lack folioles. However, when *SFT* was overexpressed in *sp* plants, a dramatic reduction in complexity was observed and leaves became progressively simple (**Figure 3**). All these features have also been obtained via graft-transmissible florigen. Thus, both flowering and the simplification of leaves reflect a shift from growth to termination via changing SFT/SP ratios.

Simplification of the compound leaves is also effective in *sp* plants, if the system is sensitized by other genes, such as *TRIFOLIATE* (*TF*). Mutant *tf* (a MYB factor, Naz et al., 2013) leaves form only one pair of leaflets (Hareven et al., 1996) and *tf* plants have enhanced apical dominance. In contrast, *tf sp* plants (high endogenous SFT/SP ratios) flower earlier and their leaves become progressively simple with the advance of flowering, mimicking *sp* plants overexpressing *SFT*. Conversely, *tf sft* leaves (low endogenous SFT/SP ratios) carry additional leaflets. However, the regular trifoliated phenotype was rescued when *sft* was inactivated as well, as in *tf sp sft*. Thus, when one of the two genes is functional, the SFT/SP ratio determines contrasting morphogenetic consequences, but as long as functional and dysfunctional *SFT* and *SP* maintain a one-to-one ratio, the morphogenetic consequences are identical (Shalit et al., 2009). This indicates that the ratio of SFT to SP, and not only their actual levels, determines the morphogenetic response. Significantly, all phenotypic changes induced by modified endogenous SFT/SP ratios, were recapitulated by graft-transmitted (systemic) florigen. In addition to rescuing the regular sympodial pattern of *sft* plants, mobile florigen, fully complemented all other pleiotropic defects of *sft*, i.e., the extended adaxial sepal, suppression of sympodial buds, defective sympodial patterning and the extra folioles, by leading to recovery of the correct SFT/SP ratios. Likewise, mobile florigen also substituted for high endogenous SFT, inducing the collapse of the sympodial pattern and the simplification of the leaves in *sp* plants, conditioning slim stems in receptor WT shoots, and stimulating leaf simplification in *tf* plants. The mobile florigen also rescued the size and structure of defective abscission zones in the floral pedicles of mutant *macrocalix* and *blind* plants (**Figure 3**; Shalit et al., 2009), and the normal flowering pattern in *miR156*-overexpressing plants (unpublished).

Are these characteristics of the SFT/SP ratio paradigm universal or unique to tomato? Accumulating observations indicate that growth and termination in reproductive and vegetative meristems in other plants are also dictated by the ratio of *SFT* and *SP* homologs. In *Arabidopsis*, termination of the inflorescence by *tfl1* is associated with simple, smaller, and more oblique leaves in long days with photoperiodic induction of FT (i.e., high FT/TFL1 ratio); all of these characteristics can be reversed when FT levels are reduced by short days, low light, cold temperatures, or their combinations (lower FT/TFL1 ratio; our observations). Suppression of flowering in the *Maryland Mammoth* tobacco grown under long-day conditions is associated with giant leaves. Likewise, the leaf complexity gradually declined in distal compound leaves of different plants approaching flowering, as seen in roses (Efroni et al., 2010). In maize, reduction of florigen or increase in SP-like (ZCN2) levels resulted in larger leaves, more branched tassels and, as in tomato, thicker stems (Danilevskaya et al., 2011). In cotton, vegetative shoots are monopodial, while reproductive fruiting branches are sympodial. High FT levels induced promiscuous arrest of the sympodial branches, small and lanceolate leaves, rather than deeply lobed leaves, and thinner stems (McGarry and Ayre, 2012a,c). In potato, florigen promoted the formation of tubers – which are determinate structures – at the tips of stolons (Navarro et al., 2011), while in grapes, misexpression of an *SP*-like gene confers a box-like architecture, instead of an inverted pear-shape architecture on Carignan fruit clusters (Fernandez et al., 2010). An *FT* homolog (not in the form of a mobile florigen) of *Populus tremula* was shown to regulate bud set and growth (Böhlenius et al., 2006). However, since *Populus* has two *FT* paralogs, with non-overlapping seasonal expression pattern (Hsu et al., 2011), it remains unknown which of the two triggers the flowering response. Finally, the significance of the SFT/SP ratio paradigm is well illustrated in the short-lived perennial *A. alpina* and long-lived perennial Rosaceae. In these cases, early and reoccurring flowering, small shoots, and simpler leaves are associated with suppression of their corresponding *SP*-like genes (Wang et al., 2011; Iwata et al., 2012; Kurokura et al., 2013).

## THE ANTAGONISTIC COMPONENT OF THE SFT/SP BALANCE

Florigen and antiflorigen are equally important constituents of floral induction (Chailakhyan, 1936a,b; Zeevaart, 1976; Lang et al., 1977). The SFT/SP ratio paradigm implies that genes belonging to the *CEN*, *TFL1*, and *SP* branch of the CETS may function as universal antagonists for florigen. But it also implies that regulated inactivation of *SFT* as such, is sufficient to generate an antagonistic developmental response.

The origin of the current florigen system in flowering plants is debated. It has been proposed that florigen endows early flowering plants, having improved conductive systems and fast-growing meristems, with faster and versatile capabilities to adapt to new habitats and respond more efficiently to different environmental signals. But because flowering is synonymous with termination and because high, unchecked florigen levels are detrimental, we speculated that the founder *SP*-like antagonist coevolved with florigen to alleviate its harmful effect (Shalit et al., 2009). Indeed, a thorough phylogenetic analysis (J. Bowman, in Shalit et al., 2009, and Supplementary information therein) showed that genes of the *CEN/TFL1/SP* branch are missing from non-flowering plants. An additional analysis confirmed these results but suggested that a duplication of an ancient *MFT* gene gave rise to an intermediate florigenic *FT*/*TFL1-like* gene in extant gymnosperms, while additional duplications gave rise to the *FT/SFT* and *CEN/TFL1/SP* branches of angiosperms (Karlgren et al., 2011). In contrast to the situation in extant gymnosperms, there are only five *CETS* in the genome of *Amborella*, the only known living species from the earliest branch in the angiosperm lineage (Amborella Genome Project, 2013): three being *MFT*-like, one a classic *FT/SFT* and one a classic *SP/TFL1* gene.

Expression of the *FT/TFL1*-like (*FTL*) genes of gymnosperms in *Arabidopsis* resulted in delayed flowering, implying a function more similar to that of *TFL-1* (Klintenäs et al., 2012). However, the temporal and spatial expression of *PaFTL2* and *PaFTL1* implied roles in late-season bud sets (Karlgren et al., 2013) and when *PaFTL2*, under a heat shock promoter, was expressed in Norway spruce (*Picea abies*), vegetative growth transitioned to terminal bud set and growth cessation, both being steps toward dormancy and winter hardiness. Therefore, despite the observations in *Arabidopsis*, the *FTL1* clade in gymnosperms functions more analogously to *FT* in angiosperms by regulating patterns of perennial growth.

Much like the odyssey of florigen (Sparks et al., 2013), the course of understanding the role of the *Arabidopsis TFL1* was quite convoluted before being determined as a universal antagonist of florigen. Mutations in *TFL1* were initially recognized because their inflorescence shoots terminated with a single flower (Shannon and Meeks-Wagner, 1991; Alvarez et al., 1992); the gene was appropriately assigned a role in maintaining the indeterminate state of the inflorescence meristem (Ratcliffe et al., 1998, 1999). But depending on growth conditions, *tfl1* also displayed modestly early flowering, condensed stem nodes and smaller and more oblique leaves (Bradley et al., 1997). Overexpression of *TFL1* induced late flowering, excessive branching of vegetative inflorescence shoots and expanded rosette leaves (Ratcliffe et al., 1998). But these features were always discussed in relation to floral and inflorescence meristem fate and to general regulation of the vegetative and reproductive phases, rather than in relation to floral transition proper. Any generalization with respect to flowering time was further hindered by the effects of the *CEN* gene of *A. majus*, which, unlike *TFL1*, did not impact flowering time when inactivated. The findings that *TFL1* forms a regulatory loop with *AP1* and *LEAFY* (Liljegren et al., 1999; Ratcliffe et al., 1999) implicated *TFL1* in regulation of the “identity” of SAMs (Ratcliffe et al., 1998). But since no place was found for *TFL1* along the linear day-length flowering pathways, its role in regulating flowering in *Arabidopsis* was largely ignored. This perspective, namely that *FT* is involved in flowering time, while *TFL1* is involved in the distinctively separate function of meristem identity, is interestingly reflected by the complete disappearance of *TFL1* from recent authoritative models and reviews on flowering (Putterill et al., 2004; Kobayashi and Weigel, 2007).

By contrast, the humble *SP* was viewed, from the outset, as a legitimate flowering suppressor and its recessive allele as a flowering-promoting gene. This was mostly due to the original assignment of *SP* as a regulator of the “determinate” vs. “indeterminate” modes of sympodial branching (Yeager, 1927; MacArthur, 1932). Like *CEN*, *SP* plays no role in primary flowering, yet, its inactivation induces accelerated termination without disrupting sympodial branching *per se*, and its overexpression delays both primary and sympodial termination. We thus regarded the gene as a legitimate component of flowering and a part of the florigen system (Pnueli et al., 1998, 2001; Lifschitz and Eshed, 2006). An important developmental consideration is that while inactivation of *TFL1*-like genes accelerates primary flowering in some plants, it always promotes flowering in secondary branches. Growing genetic and molecular evidence, from *Arabidopsis*, tomato, and other plants, provides a reasonable basis for the analysis of antagonism in the framework of the SFT/SP ratio and the endogenous and systemic functions of the *CETS* genes. Below, we consider this critical issue from various perspectives, while leaving several questions wide open.

### IT’S ALL IN THE FAMILY

*CETS* genes form a small family common to all plants, with a complexity ranging from 5 in *Amborella*, 6 in *Arabidopsis*, 12 in tomato, and 25 in maize (Danilevskaya et al., 2008, 2011). *CETS* genes belong to a family of genes encoding mammalian phosphatidylethanolamine binding proteins (PEBP; Schoentgen et al., 1987), of which the Raf kinase inhibitor protein (RKIP), which functions via direct interaction with a 14-3-3 protein is best understood (Yeung et al., 1999; Granovsky and Rosner, 2008). *CETS* have been classified into three major clades (Ahn et al., 2006; Shalit et al., 2009; Karlgren et al., 2011), named after the corresponding *Arabidopsis* genes, *TFL1*, *FT*, and *MFT* (Mother of *FT* and *TFL1*). Thus far, *MFT* genes forming the ancestral clade, have no clear relation to flowering.

Structural analysis of RKIP revealed a small [∼180 amino acids (AA)] globular protein with a putative binding pocket for phosphorylated ligands (Banfield et al., 1998; Serre et al., 1998). 3D analysis of the CEN protein confirmed the universal structural aspects of PEBPs, but also identified a unique, unstructured, external loop comprised of 14 AA (Banfield and Brady, 2000) common to all CETS proteins. Both the binding pocket and the external loop proved critical for the floral-promoting and floral-suppressing functions of *CETS* genes in *Arabidopsis* (Hanzawa et al., 2005; Ahn et al., 2006). A Y85H mutation, at the entrance of the putative binding pocket, or swapping the external loop of FT for that of TFL1, were each sufficient to convert *FT* to a flowering suppressor. However, reciprocal alterations failed to convert *TFL1* to a flowering promoter. In addition, the 14-AA external loop has been shown to be conserved among *FT*-like genes, while those of *TFL1* and *MFT*-like genes are widely divergent. A recent comprehensive study identified only four additional AA that were critical for converting *FT* to a functional *TFL1* (Ho and Weigel, 2014).

Natural conversion of an *FT*-like gene to a floral suppressor was found in domesticated sugar beet, where a single Y to N conversion in the external loop of an FT-like paralog generated an FT antagonist (Pin et al., 2010). It will be interesting to explore the role of the authentic *SP/TFL1*-like gene in this species. However, as more *CETS* genes and their functions in different plants are reported (Meng et al., 2011; Hecht et al., 2011), it is becoming increasingly difficult to specify the number of AA alterations required to provide the external loop with a universal repressing role. Interestingly, *CEN* and *SP* independently antagonize flowering in *Arabidopsis*, but *TFL1* is inert in tobacco and tomato (Amaya et al., 1999; Ahn et al., 2006). As *CETS* genes can counteract florigen via various mechanisms, sequence-based functional predictions can be misleading by missing the critical residues and the underlying mode of action.

An additional mechanism, generating antagonistic *FT*-like gene products by posttranslational modification may involve the ligand binding pockets. Nakamura et al. (2014) reported the association of phosphatydilcholine by the ligand-binding pocket of FT, suggesting the possibility other phosphorylated ligands, or a yet unknown post-translation modification, function as auto-antagonists.

In tomato, tobacco, and *Arabidopsis*, the natural systemic antiflorigens candidates were *SP*, *CET2/4*, and *TFL1*, respectively (Zeevaart, 2006), genes that, unlike *FT* paralogs, are preferentially expressed in the SAM. Short-range, cell-to-cell movement via plasmodesmata was shown for TFL1 in *Arabidopsis* (Conti and Bradley, 2007) and such movement may be a general feature of *CETS* genes. Nevertheless, it was recently reported that one of two *TFL1-like* genes in *Chrysanthemum seticuspe* expressed in leaves, *CsAFT*, suppresses flowering under inductive short-day conditions, and also induces late flowering via grafting (Higuchi et al., 2013). It is possible that in other species, other antagonistic CETS are preferentially expressed in leaves and systemically translocated to the apex. In tomato, *SP* is predominantly expressed in the apex, but *SP5G*, a potential promoter of indeterminacy, is predominantly expressed in the leaves. Thus, while florigen was the first protein hormone characterized in plants, other members of the family, being functional homologs or antagonists, are also likely to function at long range.

### CETS PROTEINS FUNCTION AS PARTNERS IN TRANSCRIPTION COMPLEXES

The most likely mechanism by which *TFL1*-like genes antagonize their flowering-promoting homologs is via formation of competitive or antagonistic transcription complexes. The first candidates for a core functional complex of CETS factors were discovered as SP-Interacting Proteins (SIP, Pnueli et al., 2001), and included the 14-3-3 adaptor proteins, SPGB (SP-associated G-box), a bZIP transcription factor homolog of FD, an SP-associated NIMA-like kinase (SPAK, Osmani et al., 1988), and a 99-amino acid-long polypeptide called SIP4. All SIPs independently interacted with the 14-3-3s and in addition, SPGB interacted with the *Arabidopsis* FT. SP and FT bind different 14-3-3s with different affinities and SPAK and SIP4 are, most likely, SP-specific (Pnueli et al., 2001). The plasticity, flexibility, and diversity of protein–protein interactions suggest that CETS proteins, in analogy with the 14-3-3 adaptors, function as hubs in signaling systems, with the potential to integrate a wide variety of environmental cues (Pnueli et al., 2001).

In a recent milestone discovery, Taoka et al. (2011) presented a 3D structure of the hetero-hexameric complex consisting of two molecules each of the rice FT homolog (Hd3a), 14-3-3 and FD (OsFD1), and named it florigen activation complex (FAC). In this assembly, the 14-3-3 protein forms a bridge between Hd3a and OsFD1, which otherwise do not directly interact. It was therefore postulated that the 14-3-3s are the primary intercellular receptors for mobile florigen, and that the successful interactions between bZIPs (FD clade) and FTs in yeast are actually mediated by BMH1 and BMH2, the two endogenous yeast 14-3-3s. But, as logical as these conjectures are, direct experimental support for both is lacking. Taoka et al. (2011) reported that other proteins, such as KANADI and WAVE-DAMPENED2 homologs, which share the SAP (Ser-Ala-Pro) domain required for binding with 14-3-3s, also recognized Hd3a. We identified a calmodulin binding protein of the IQD type (Abel et al., 2005), which similarly interacts with 14-3-3 proteins in a similar fashion. Ho and Weigel (2014) discovered several TCP transcription factors that differentially interact with FT and TFL1. However, different TCP binding partners were reported for the same *Arabidopsis* CETS proteins (Liu et al., 2012; Hiraoka et al., 2013; Ho and Weigel, 2014). In our experiments, SP interacted with a TCP (AF175965) that was later shown to be encoded by *LANCEOLATE* (Ori et al., 2007) and similar TCP factors suspiciously interacted with BELL, KN, or CO. Thus, the relevance of TCB, and similarly the specificities and redundancies of the 14-3-3s to the regulatory functions of florigen require further study.

Both SFT and SP of tomato bind in yeast 14-3-3 and bZIP (SPGB) proteins, but also have their own specific binding proteins (Pnueli et al., 2001). In *Arabidopsis*, FT and TFL1 bind FD and FDP (Abe et al., 2005; Wigge et al., 2005), but the intercellular localization of TFL1 is questionable (Conti and Bradley, 2007; Sohn et al., 2007). The likely inference is that antagonistic proteins of the CETS family compete for a position in the same core functional complexes, or that stoichiometric relations permit the formation of two overlapping but nonidentical transcription complexes. Under either condition, the two complexes may compete for common targets or each may activate distinct antagonistic processes.

### TARGET COMPETITION

One candidate target system for FT-TFL1 (SFT-SP) antagonism involves the *FT-*targeted *AP1*-*SOC1-LEAFY* network, which is partially regulated by *TFL1* (Ratcliffe et al., 1999) and activates inflorescence and floral identity genes (Kobayashi and Weigel, 2007; Turck et al., 2008). However, the definition of *AP1* as a primary target of *FT* and a pivotal flowering gene is problematic. Schmid et al. (2003) showed that activation of *AP1* by long days is delayed until after *SOC1* and *FUL* have responded. Yamaguchi et al. (2009) assigned the *AP1* output to the meristem identity rather than to the reproductive transition. High activity of *LFY*, *AP1*, or even *FUL* indeed induce early flowering, but it is equally important to note that, unlike *ft* or *soc1*, inactivation of *LFY* or *AP1* has no, or only a marginal, effect on flowering time (Ruiz-García et al., 1997). Likewise, in tomato, inactivation of *FALSIFLORA (FALS)*, the *LEAFY* homolog, or *MACROCALYX* (*MC*), the *AP1* homolog, does not appreciably delay flowering. Significantly high *SFT* levels are epistatic to the “vegetative” genes *fals*, *mc*, and *j1* and to all their double combinations [*J1*, *JOINTLESS* is a *MADS* gene which interacts genetically with *SP* (Pnueli et al., 1998; Szymkowiak and Irish, 2006) and the resulting early terminating organs maintain the authentic mutant phenotypes (Shalit et al., 2009)]. While it is still unknown if constitutive overexpression of *FT* is epistatic to the various combinations of *ap1* with the *soc1* and *agl24* late-flowering mutants, flowering of *soc1 agl24* double mutants is delayed in short days (Li et al., 2008). Additional modifications in the current schemes for flowering transition are required to include the phenotypic interactions of *svp* with the double suppressor of flowering *soc1-2 ful-2*, as reported by Torti et al. (2012).

Additional inconsistencies in present models must be considered too. *fdp*, *fd,* or *fd fdp* double-mutant *Arabidopsis* plants flower, where the double mutant flowering is not as late as in *ft* and certainly not as late as in *ft tsf* (Yamaguchi et al., 2005; Jaeger et al., 2013). Tomato *sft* plants also flower but, unlike with *ft*, full proof null alleles are not available. Interestingly, unlike *Arabidopsis* or wild tomato species, the cultivated tomato genome carries no other functional *SFT* homologs.

Obviously, difficulties in reaching a coherent model arose from the ever-increasing complexity of the genetic interactions and from the lack of clarity at numerous levels: the relations between termination, inflorescence specification, and floral differentiation, between phase transition and meristem fate and between determinate and indeterminate shoots. In the indeterminate and monopodial shoots of *Arabidopsis*, only axillary meristems terminate to form flowers, whereas in tomato, the determinate and sympodial meristems themselves terminate and subsequently differentiate to form an inflorescence. Further understanding requires that the molecular interactions of the systemic flowering antagonists with the FT-dependent MADS target network be properly appreciated. Furthermore, to fully understand the florigen hormone and the SFT/SP regulatory hierarchy, their end users, i.e., cellular systems such as cell cycle, or cytoskeleton, not just transcription networks, should be identified.

## GENERAL CONSIDERATIONS RELATING TO THE SYSTEMIC PATHWAY OF FLORIGEN

Classic grafting experiments supported the hypothesis that florigen uses the phloem track to move from leaves to the shoot apices (Zeevaart, 1962, 1976). This premise acquired molecular support when *CO*, regulated by companion cell-specific promoters, *AtSUC2* from *Arabidopsis* and *CmGAS1* of *Cucumis melo*, successfully induced flowering under non-permissive conditions (An et al., 2004; Ayre and Turgeon, 2004). It was also shown that *FT* is predominantly expressed in the vasculature and that FT-derived polypeptides were detected in the phloem sap of several species. It should be noted that three phloem tracks (primary, secondary and an additional centrally located phloem) are open for the mobile florigen in Solanaceae and Cucurbitaceae (Esau, 1969), but no particular mechanistic preference has been reported. At the cellular level, most tagged FT and SFT proteins were located in the nucleus (Abe et al., 2005; Lifschitz et al., 2006), but the cytoplasmic compartments hosting the rest of the protein were not identified.

Reciprocal grafting experiments in tomato demonstrated that, in these plants, florigen moves upward and downward from *SFT*-overexpressing source shoots, and enters axillary buds (sinks) of the recipient stems, as was inferred from the classic analysis of florigen (Zeevaart, 1976). In a standard grafting experiment in tomato, a donor scion overexpressing *SFT* is grafted onto a non-flowering *sft uf* (*uniflora*, Dielen et al., 2004) recipient tester with 3–4 basal leaves and their transiently dormant axillary buds. Flowering is then recorded in the out-growing laterals of the non-flowering tester (stalk). To induce flowering in this setup, florigen must translocate from the donor leaves of the scion downward to the activated axillary shoots. In a reciprocal graft, florigen is moved upward from leaves of the donor stalk to organs and apices of the recipient *sft uf* scion.

The cellular mechanisms regulating the intercellular migration of florigen have not yet been ascertained. As a globular protein within the size exclusion limits of plasmodesmata, florigen may enter and exit the translocation stream by a non-selective process. Recently, a newly discovered FT-interacting protein (FTIP1; Liu et al., 2012), which is associated with the endoplasmic reticulum, was suggested to mediate the exit of FT from phloem companion cells to sieve elements in *Arabidopsis*. However, the impact of FTIP1 on flowering is relatively mild and, more importantly, it does not bind TFL1. If the claimed specificity of FTIP1 (Liu et al., 2012) holds true, it follows that each CETS protein may require its own exit chaperon. Recently, experiments combining *FT*-mutant genes, grafting tests, and a viral expression system in *Cucurbita moschata*, identified mutant FT variants that cannot exit the phloem. It was suggested, that the FT protein enters the phloem translocation stream through either the selective or non-selective pathway. But active uploading from the phloem to the (unknown) target cells in the SAM (Yoo et al., 2013) requires short-distance active cell-to-cell movement, via plasmodesmata.

In *Arabidopsis*, *FD* is expressed exclusively in the SAM (Abe et al., 2005; Wigge et al., 2005) and it has therefore been rightfully accepted, that florigen must reach the apex in order to induce flowering. However, due to low levels of the traveling protein and poor resolution, direct evidence for a tagged mobile florigen reaching the target cells in the SAM proper remains to be obtained. All reported cases, in *Arabidopsis*, rice, or potato, recorded florigen close to, but not in, the SAM (Corbesier et al., 2007; Tamaki et al., 2007; Navarro et al., 2011). Another line of evidence, namely that FT proteins are found in the phloem sap, is required but conceptually problematic. This is because thousands of proteins have been detected in the phloem sap of several species but, with the exception of few, their functional significance in this critical compartment is poorly understood.

Interestingly, in tomato, all members of the *FD* clade are expressed in leaves and at levels higher than *SFT* and in addition florigen enter leaves in which *SFT* is already expressed (Shalit et al., 2009). If, as we propose, the critical impact of florigen is on regulation of preexisting SFT/SP ratios in all organs, it is likely that, in some species, *FT-like* genes are also expressed in the SAM to maintain a local pre-flowering ratio as in tomato leaves. Indeed, we found that *SFT* is expressed in the primary SAM of tomato from its early inception (A. Shalit, personal communication).

Florigen was the first protein in plants demonstrated to function long range and to promote growth attenuation in all above-ground meristems, with flowering being its most visual output. The reiterated phase transition, the perennial evergreen nature of the tomato shoot system and the day-neutral flowering response require that the distribution of, and the response to florigen, be regulated on a daily basis. Mature leaves are the primary source of florigen and the removal of young tomato leaves accelerates flowering (Leopold and Lam, 1960). Florigen produced by the mature parts of the compound leaf is distributed to younger leaflets and subsequently to all organs, including leaves where *SFT* is already expressed. Thus, on the whole-plant level, by regulating leaf maturation, florigen controls its own levels and mode of distribution (Shalit et al., 2009).

## CONCLUSION

While the core SFT/SP ratio paradigm initially provided a conceptual framework for the analysis of the growth versus termination equilibrium in the reproductive and vegetative meristems of tomato, supporting observations from other flowering systems conferred it broader and more general significance. It presents the best platform for further exploration of the seemingly unrelated vegetative/reproductive phenomena in several plants.

The complex interactions among *CETS* genes, the rapid expansion and decline in the size of this gene family, and their species-specific incorporation into environmental sensing-programs, have facilitated evolution of a range of flowering modes, from simple, fast-cycling annuals to complex perennials, where different branches display different autonomous flowering modes.

Transition to flowering, inflorescence differentiation, and leaf morphogenesis are quantitative and cumulative processes. The cellular mechanisms underlying the response of meristems to changing SFT/SP ratios remain unknown and it is still premature to determine the basis for growth/termination, not only vegetative/reproductive, shifts, in each meristem, or more so, in different flowering plants. Notably, the dramatic enlargement and doming of the vegetative apical meristem presents a common indication of floral transition (Bernier, 1988). Thus, SFT/SP targets must include regulators that modify the rate and orientation of cell divisions enforcing a redistribution of local signaling systems.

Adaptation of plants to different environments, an obvious consequence of domestication by humans, requires changes in flowering regulation, as well as in shoot architecture, with an unpremeditated exploitation of the SFT/SP ratio playing a seminal role in this process. As described here, these attributes were first recognized by the exploitation of a mutant *SELF PRUNING* gene, where accelerated termination of the WT regular sympodial cycles facilitated rapid breeding of mechanically harvested tomatoes (Rick, 1978; Pnueli et al., 1998). In retrospect, the same genetic system was exploited during the domestication, via selection of naturally occurring CETS alleles, to revolutionize a wide range of crop plants, or even to turn an exotic plant into a crop. These include, in addition to tomato, direct exploitation of changes in SP-like genes in soybeans (Tian et al., 2010), beans (Kwak et al., 2008), roses, strawberries (Iwata et al., 2012), or even in barley (Comadran et al., 2012). Likewise, mutations converting duplicated *FT*-like genes into floral antagonists were selected during domestication of sunflowers (Blackman et al., 2010), and sugar beet (Pin et al., 2010). Allelic variations in *SFT/FT*-like genes or in their upstream regulation have been reported for rice (Kojima et al., 2002; Ogiso-Tanaka et al., 2013), wheat (Bonnin et al., 2008), and potato (Kloosterman et al., 2013). Tuning of the SFT/SP ratio constitutes one of the most important processes behind crop domestication.

## Conflict of Interest Statement

The authors declare that the research was conducted in the absence of any commercial or financial relationships that could be construed as a potential conflict of interest.
